# The transition between acute and chronic infections in light of energy control: a mathematical model of energy flow in response to infection

**DOI:** 10.1098/rsif.2022.0206

**Published:** 2022-06-22

**Authors:** Gang Zhao, Rainer H. Straub, Michael Meyer-Hermann

**Affiliations:** ^1^ Department of Systems Immunology and Braunschweig Integrated Centre of Systems Biology, Helmholtz Centre for Infection Research, Rebenring 56, 38106 Braunschweig, Germany; ^2^ Laboratory of Experimental Rheumatology and Neuroendocrine Immunology, Department of Internal Medicine, University Hospital Regensburg, 93042 Regensburg, Germany; ^3^ Institute for Biochemistry, Biotechnology and Bioinformatics, Technische Universität Braunschweig, Braunschweig, Germany

**Keywords:** energy, inter-organ interactions, infections, immune response, lipolysis resistance, ageing

## Abstract

Background: Different parts of an organism like the gut, endocrine, nervous and immune systems constantly exchange information. Understanding the pathogenesis of various systemic chronic diseases increasingly relies on understanding how these subsystems orchestrate their activities. Methods: We started from the working hypothesis that energy is a fundamental quantity that governs activity levels of all subsystems and that interactions between subsystems control the distribution of energy according to acute needs. Based on physiological knowledge, we constructed a mathematical model for the energy flow between subsystems and analysed the resulting organismal responses to *in silico* infections. Results: The model reproduces common behaviour in acute infections and suggests several host parameters that modulate infection duration and therapeutic responsiveness. Moreover, the model allows the formulation of conditions for the induction of chronic infections and predicts that alterations in energy released from fat can lead to the transition from clearance of acute infections to a chronic inflammatory state. Impact: These results suggest a fundamental role for brain and fat in controlling immune response through systemic energy control. In particular, it suggests that lipolysis resistance, which is known to be involved in obesity and ageing, might be a survival programme for coping with chronic infections.

## 1. Introduction

Brain, gut and immune system are part of a complex network with interactions mediated by molecules, cells and wired connections [1–3]. Knowledge concerning interactions in this complex network is accumulating, having reached a level that challenges holistic understanding. Energy is needed in all vital subsystems to retain functionality and maintain organ integrity. Accordingly, energy homeostasis is tightly regulated both locally (within-organ level) and globally (trans-organ level). Excess energy is stored in fat tissue and can be released upon increased needs, by mechanisms such as *β*-adrenergic stimulation of lipolysis [4]. Increased needs are signalled in response to various physiological stresses, for example, immune responses to invading pathogens. We have developed a mathematical model of energy flow between five main vital compartments, thus aiming to explore the potential synergisms and/or energy trade-offs between organs, to provide an holistic understanding of the whole network.

Mathematical models can help in understanding the functional interdependence of the network of interacting subsystems of an organism. Such network models have been developed at various scales, such as genetic networks [5], molecular networks connecting different subsystems [6], networks of cellular trafficking between organs [7] and multi-organ networks [8] up to population levels [9]. The systems biology community has developed theories and software tools that incorporate energy conservation laws into biochemical networks based on thermodynamic principles [10–13]. At the same time, mathematical models of metabolic energy regulation, with complexities ranging from simple glucose control loops to neural-endocrine control of appetite involving various kinds of nutrients, have been extensively studied in the field of diabetes and obesity [14–17], as well as in sports physiology [18].

To the best of our knowledge, energy was never a network-focus of interacting organismal subsystems. Here, interactions between organs/tissues are formulated in terms of energy flow, where competition, synergisms and trade-offs on the energy-flow level have been naturally translated to the functional level of participant organs/tissues. Our working hypothesis is that dysregulation of energy homeostasis, and the subsequent trade-offs between various organs/tissues, might explain pathogenesis of various systemic diseases, and reveal a potential advantage of this modelling approach over traditional biomolecular/cellular pathway-based modelling.

We have illustrated the concept by drawing on the example of energy flow adaptation induced by immune responses. Given that an immune response, in particular, the acute phase response, is usually associated with anorexia of various degrees, it is expected that sufficient energy stored in fat tissue or supplied environmentally by food is fundamental to success [19], and that the highly energy-dependent inflammation must cease within several weeks to prevent long-term deterioration [20].

However, chronic inflammation is ubiquitous and has emerged as a shared factor in a variety of diseases, notably type 2 diabetes, cardiovascular disease, chronic obstructive pulmonary disease, cancer, asthma, Alzheimer's disease and autoimmune diseases like rheumatoid arthritis, multiple sclerosis and systemic lupus erythematosus [20,21]. Moreover, inflammatory pathways and their associated molecules are continually being identified as reliable markers of ageing, and as risk factors for diseases associated with ageing [22–24]. Chronic inflammation, whether occurring in the absence of overt infection or due to latent pathogens, leads to immune senescence [25,26]. There is also growing mechanistic evidence that chronic inflammation both accelerates and is exacerbated by systemic ageing processes [27–30]. Although growing fast, our current understanding of the precise aetiology of chronic inflammation, as well as its role in immunity and obesity throughout lifespans, remains woefully insufficient [31,32].

The energy-based modelling work presented in this paper allowed us to reproduce common systemic behaviour in acute infections. Moreover, we were able to formulate conditions for the transition between acute and chronic infections revealing a hitherto unappreciated role played by fat tissue.

## 2. Results

### 2.1. A model of energy flow

**Figure 1.**
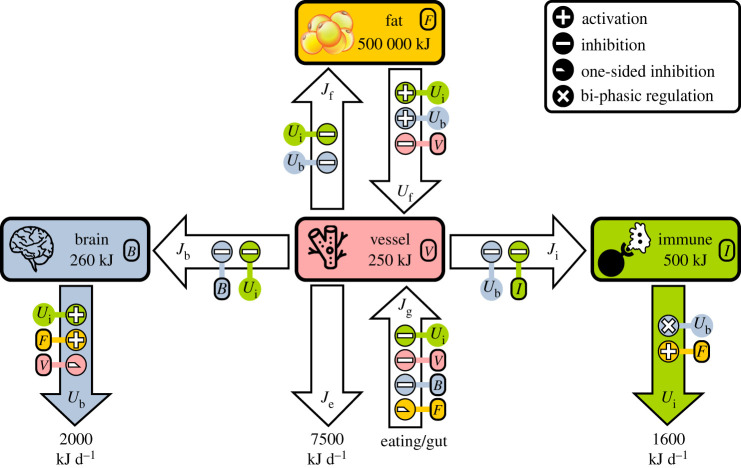
Schematic of the energy-flow model. The four compartments of the network comprise brain (*B*), immune system (*I*), blood vessel (*V*) and fat tissue (*F*). Energy enters the system from the gut (*J*_g_) for use by the brain (*U*_b_), immune system (*U*_i_) and other tissues (*J*_e_). Fat tissue acts as an energy buffer and blood vessels as a central connector of these compartments. The amount of usable energy in the brain and immune system undercuts their daily usage even under healthy conditions, indicating that these two organs largely rely on energy flow from fat (*U*_f_) or gut (*J*_g_) to blood (*V*), and subsequently from blood to the brain (*J*_b_) and to immune system (*J*_i_). Energy flow between compartments is regulated by network variables (symbols linked to signs in the legend; colours depict the regulating compartment). One-sided inhibition operates only when the regulating factor is below its homeostatic level. See text for details. Numbers indicate homeostatic levels of energy in kJ or energy flow in kJ d^−1^.

Energy flow in a network of four connected human organismal compartments was formulated in the simplest possible manner, albeit strictly based on known physiological principles (figure 1; electronic supplementary material, Methods):

(1) Stress (*U*_b_) and immune (*U*_i_) responses (units in kJ d^−1^) regulate energy release from fat by lipolysis (*U*_f_, units in kJ d^−1^) [33], and energy uptake (*J*_f_, units in kJ d^−1^) by modulation of insulin secretion [34] and resistance [35].
(2) Brain and immune system are mutually regulatory: the immune response (*U*_i_) triggers the HPA axis (*U*_b_) [36] and limits energy uptake by the brain (*J*_b_, units in kJ d^−1^) [37]. HPA axis activation (*U*_b_) leads to cortisol release, which has profound inhibitory effects on the immune system (*U*_i_ and *J*_i_, units in kJ d^−1^), such as suppressing the activities of key signalling molecules and transcription factors in inflammatory pathways and inducing thymocyte apoptosis [38]. However, the sympathetic nervous system and HPA axis (*U*_b_) are also pro-inflammatory in specific contexts [38], especially when briefly activated to an intermediate level [39], implying biphasic regulation of *U*_i_ by *U*_b_.
(3) Homeostatic self-regulation of energy in brain (*B*, units in kJ), immune system (*I*, units in kJ) and blood vessels (*V*, units in kJ): these include the effect of *B* on *J*_b_, *I* on *J*_i_, and *V* on *U*_f_ and *J*_g_ (units in kJ d^−1^). This reflects homeostatic mechanisms, such as regulation of glucose transporters, on the cell membrane by cytoplasmic ATP [40], of insulin and glucagon secretion in the endocrine system, and of eating regulated by blood glucose [34].
(4) Fat tissue homeostasis by indirect regulation of other compartments, comprising (i) fat (*F*, units in kJ) regulation of brain energy usage (*U*_b_) via afferent sensory nerve fibres [41]; (ii) fat (*F*) regulation of immune energy usage (*U*_i_), for example via leptin [42,43]; and (iii) lack of fat energy (*F*) increases eating behaviour (*J*_g_) via leptin, which, in turn, signals negative energy balance and decreased energy stores (one-sided inhibition) [44].
(5) Brain and immune system request energy upon stimulation: the brain (*B*) ensures energy supply from blood vessels by regulating appetite and digestion (*J*_g_) [34]. At low energy in blood vessels (*V*), a brain response (*U*_b_) is initiated (one-sided inhibition), which then increases energy flow from fat tissue to blood vessels [45,46]. The immune response (*U*_i_) blocks energy expenditure for foraging (*J*_g_) by cytokine-induced anorexia and fatigue [47].

Homeostatic levels of usable energy in each compartment were estimated by considering various forms of biomolecules (carbohydrates, lipids, ketone bodies, etc.) in blood, and by assuming a linear relationship between the energy level and the mass of the organ/tissue. Homeostatic energy flow was estimated based on published values. Regulation of energy flow was modelled parsimoniously by assuming the simplest form. Refer to the electronic supplementary material, methods, for a detailed explanation of the model.

### 2.2. Response to infections and therapies

**Figure 2.**
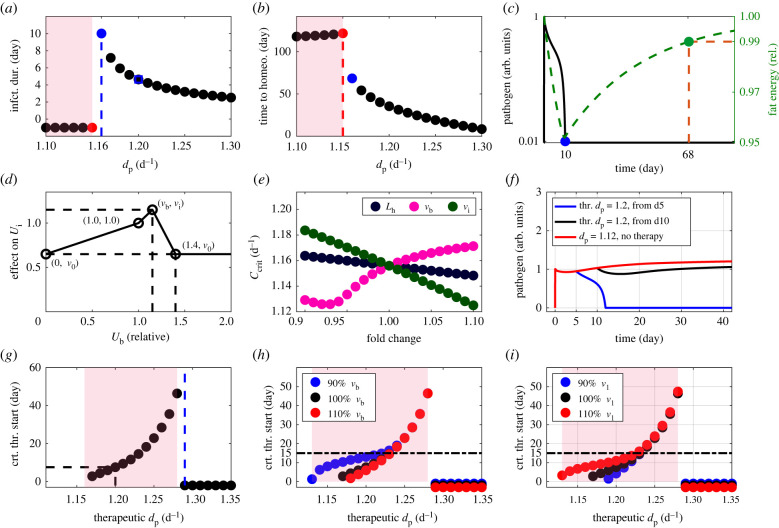
Acute versus chronic infections in the energy network. Starting from network equilibrium, pathogen was raised from 0 to *P*_0_ = 1 (see electronic supplementary material, equation S3) at day 0. (*a*) Time until pathogen elimination (1% of inoculation dose) in dependence on the clearance rate (*d*_p_). Negative values denote persisting pathogen. A critical clearance rate (*C*_crit_) for pathogen elimination exists between the red and the blue points. (*b*) Time until energy homeostasis (healthy or diseased) is re-established to 99% in all compartments. (*c*) Pathogen dynamics (black, left axis) and fat energy content (green, right axis) with a clearance rate (*d*_p_) at the blue point in (*a*). (*d*) Assumed biphasic impact of brain activity on the immune response (figure 1; *U*_b_ regulates *U*_i_). The healthy homeostatic state assumed to be (1.0, 1.0). Maximum stimulation is at (*v*_b_, *v*_i_), with *v*_0_ the maximum inhibition. The physiological upper limit of the brain energy usage rate is 1.4-fold (see Methods). (*e*) The critical clearance rate (*C*_crit_) in dependence on the most sensitive host properties (*d*_p_ fixed, blue square in *a*). (*f*) Pathogen level in response to a therapy raising the clearance rate from *d*_p_ = 1.12 d^−1^ to a therapeutic *d*_p_ = 1.20 d^−1^ (blue square in *a*) from day 5 (blue) or day 10 (black) or not applied (red). (*g*) The critical time *T*_c_ to start the therapy that clears the pathogen in dependence on the therapeutic *d*_p_. Negative values mean that the therapy can be started at any time. (*h*,*i*) Impact of modulation of *v*_b_ and *v*_i_ by + 10% (red) and −10% (blue) on the critical time *T*_c_ to start a successful therapy (reference simulation (black) with parameters in electronic supplementary material, table S1). The light pink background indicates the regime of chronic infection in the case of no therapy (*a*,*b*) or when the therapy is applied after the critical time *T*_c_ (*g*,*h*,*i*). infct. dur., infection duration; time to homeo., time to homeostasis; arb., arbitrary;thr*.*, therapeutic; crt*.*, critical.

At first, we challenged the energy flow model at its healthy homeostatic state with a one-time pathogen dose, subsequently inducing competition between pathogen growth and pathogen clearance by the immune response (*U*_i_). The pathogen clearance rate (*d*_p_) determined the duration of the infection (figure 2*a*), as well as the time required to re-establish homeostasis (figure 2*b*). Below a critical pathogen clearance rate (*C*_crit_; see electronic supplementary material, figure S1), the pathogen persisted and the infection became chronic (figure 2*a*, negative values). Thus, the model captures acute and chronic infections.

In the acute infection regime, a higher pathogen clearance rate (*d*_p_) was associated with shorter infection duration (figure 2*a*) and with a faster return to homeostasis (figure 2*b*). The infection was associated with weight loss and the fat compartment started a slow recovery only after pathogen clearance (figure 2*c*). In this example, the pathogen was cleared at day 10 while the fat compartment recovered in more than two months (figure 2*c*). In the chronic infection regime, the system reached a new steady state associated with a persistent pathogen load. This transition was slow (figure 2*b*, left of the red point), where higher pathogen clearance rates (*d*_p_) were associated with lower homeostatic pathogen load (electronic supplementary material, figure S1A) and longer time to reach homeostasis (figure 2*b*).

Because a lower critical clearance rate (*C*_crit_) indicates facilitated pathogen clearance, we next investigated how this depends on pathogen and host properties (electronic supplementary material, figure S2). The pathogen replication rate (*r*_p_) and the carrying capacity (*P*_max_) were the most sensitive pathogen properties (electronic supplementary material, figure S2E,F). Higher values increased infection duration and *C*_crit_. Part of the host factors are parameters *v*_b_, *v*_i_ and *v*_0_ (defined in figure 2*d*) for the bimodal effect of the brain stress response (*U*_b_) on the immune response (*U*_i_). A sensitivity analysis identified *v*_i_ and *v*_b_ and the homeostatic lipolysis rate (*L*_h_, see electronic supplementary material, equation S15) as most important for *C*_crit_: higher *L*_h_ and higher *v*_i_ both led to lower *C*_crit_ (figure 2*e*) and shorter infection duration (electronic supplementary material, figure S2A). The impact of *v*_b_ was biphasic (figure 2*e*), consistent with the existence of an optimal brain stress response for most efficient immunity [48]. The optimal *v*_b_ (figure 2*e*, magenta, minimum) was modulated by *L*_h_ and *v*_i_ (electronic supplementary material, figure S2C,D). These results suggest that the brain stress response and lipolysis are host parameters critical for pathogen clearance.

Starting from a chronic infection (figure 2*f*, red, *d*_p_ = 1.12 d^−1^), we investigated the effect of a therapy that boosts the pathogen clearance rate (*d*_p_) to a higher ‘therapeutic *d*_p_' (*d*_p_ = 1.20 d^−1^). An identical therapy resolved the infection when applied from day 5 (figure 2*f*, blue), but not if applied from day 10 onwards (figure 2*f*, black). In this example, the critical time to start a successful therapy was 8 days post-infection (figure 2*g*, dashed black line). The emergence of a critical therapy initiation time is related to dynamic modulation of immune energy usage (*U*_i_) due to interactions with other network compartments. This critical time was longer for stronger therapies (figure 2*g*), suggesting that weak therapies must be applied early to ensure pathogen clearance. At a minimal therapeutic strength (figure 2*g*, blue vertical line), clearance was achieved irrespective of its initiation time (figure 2*g*) while minimizing (potential) side effects.

For weak regimens, critical therapy initiation time (*T*_c_) depended on the impact of brain stress on the immune response (*v*_b_ and *v*_i_ in figure 2*d*). These two parameters exhibited considerable and opposing impacts on *T*_c_ (figure 2*h,i*). For strong therapies associated with longer critical times, their impact was negligible and the parameter controlling maximum inhibition of the immune response by brain stress (*v*_0_) became important (electronic supplementary material, figure S3A). The effect of the homeostatic lipolysis rate (*L*_h_, see electronic supplementary material, equation S15) was twofold: higher values prolonged the critical time when less than 15 days, and shortened it otherwise (electronic supplementary material, figure S3B). The impact of other host factors on the critical therapy initiation time was small (electronic supplementary material, figure S3C). These results suggest that after 15 days of infection, the organism switches its mode of coping with persistent infections.

**Figure 3.**
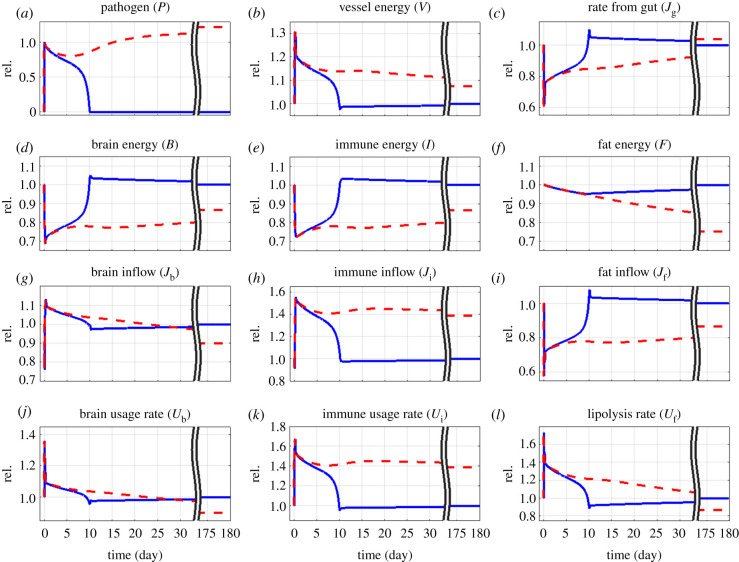
Model response to acute (blue) or chronic (red) infections. Starting from network equilibrium, pathogen level was raised from 0 to *P*_0_ = 1 (see electronic supplementary material, equation S3), at day 0. *d*_p_ = 1.16 d^−1^ (blue, acute infection) and 1.15 d^−1^ (red, chronic infection), see blue and red points in figure 2*a*. All quantities are defined in figure 1 and electronic supplementary material, equations S3–S14, and normalized with respect to their healthy homeostatic levels (rel.). Parameters are given in electronic supplementary material, table S1. The broken time axis indicates long-term behaviour.

Next, we further characterized energy dynamics in response to infections in network compartments. In the course of acute *in silico* infections (figure 3, blue curves, corresponding to the blue point in figure 2*a*), energy in blood vessels was transiently upregulated (figure 3*b*), while energy flow from gut was suppressed (figure 3*c*), reflecting increased serum levels of fatty acids [49] and anorexia [50] during acute infections, respectively. Energy in the brain was transiently reduced to less than 70% (figure 3*d*) but recovered after pathogen clearance, corresponding to acute infection-induced sickness behaviour [37].

Although energy in the brain and the immune system showed similar dynamics (figure 3*d,e*), increased energy inflow and usage was more pronounced and prolonged for the immune response (figure 3*g,j* versus 3*h,k*). The inflow of energy into fat tissue was downregulated (figure 3*i*) while outflow was upregulated (figure 3*l*), consistent with its role as the energy supplier during infections in a state of acute anorexia [51]. The extent of energy loss in fat tissue (figure 3*f*) was less than 5%. Recovery was slow (figure 3*f*, see also figure 2*c*) and relied on slowly increasing appetite (figure 3*c*) when brain and immune usage rates returned to normal (figure 3*j,k*) and their energy content restored (figure 3*d,e*). The overshoot in energy uptake in gut, brain and immune system at day 10 (figure 3*c–e*) was required to return to healthy homeostasis. This is also reflected in the fat turnover (figure 3*i,l*) which compensates loss of fat energy. Overall, the model was consistent with known physiologic dynamics in response to acute infections [52].

In chronic *in silico* infections (figure 3, red dashed lines, corresponding to the red point in figure 2*a*), the network was driven into a new homeostatic state—that associated with inflammation. Compared with the healthy state, the diseased state after 175 days of infection was associated with increased energy in the vessel compartment (figure 3*b*), slightly increased energy inflow from gut (figure 3*c*), reduced brain and immune system energy (85% of the healthy state; figure 3*d,e*), and reduced fat energy (75% of the healthy state; figure 3*f*).

Transition from the acute fight with the pathogen to the state of chronic coexistence was characterized by a rebound in the pathogen load at day 6 (figure 3*a*; electronic supplementary material, figure S4), concomitant with a transient and attenuated increase of the immune response (figure 3*k*; electronic supplementary material, figure S4A) and a steady decrease in the brain stress response (figure 3*j*; electronic supplementary material, figure S4B). The relationship between pathogen load and immune response switched around day 18 from a positive to a negative correlation (electronic supplementary material, figure S4A) indicating relative weakness of the immune system after day 18. Energy in the brain and immune system started to recover after day 18 (electronic supplementary material, figure S4C,D), indicating a trade-off between pathogen clearance and organ integrity [53]. By contrast, fat energy decreased continuously before reaching the new disease-related homeostatic state.

### 2.3. Effects of lipolysis resistance

**Figure 4.**
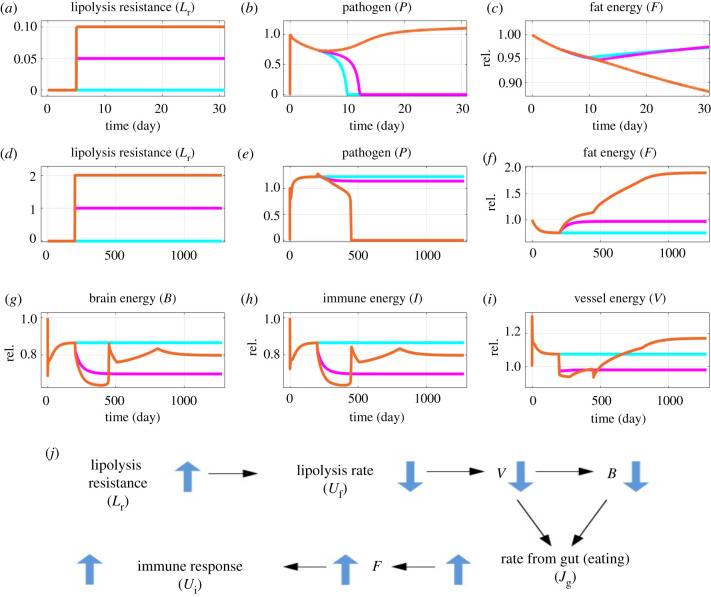
Lipolysis resistance (LR) impairs acute, but improves chronic immune response. Starting from network equilibrium, pathogen level was raised from 0 to *P*_0_ = 1 (see electronic supplementary material, equation S3), at day 0. (*a–c*) In an otherwise cleared infection (*d*_p_ = 1.16 d^−1^; blue point in figure 2*a*), LR was raised from 0 to 0.05 (magenta) or 0.1 (orange) from day 5 onwards (*a*). This delayed or prevented pathogen clearance (*b*). Fat energy was reduced (*c*). (*d–i*) In chronic infection (*d*_p_ = 1.15 d^−1^; red point in figure 2*a*), LR was raised from 0 to 1 (magenta) or 2 (orange) from day 200 onwards (*d*), which reduced or cleared the pathogen (*e*) and impacted fat (*f*), brain (*g*), immune (*h*) and vessel (*i*) energy. The cyan curves show the reference *L*_r_ = 0. The cusps in the curves before day 500 (*g–i*) reflect rapid dynamics around pathogen clearance. The left side of the cusp is associated with rapid pathogen clearance (*e*), the right with increased fat energy (*f*). The inflection points after day 500 (*g,h*) are due to the inflection points in the biphasic effects of the stress hormone axes responses onto the immune system (see also figure 2*d*). (*j*) Mechanisms involved in LR improved immune response: the increase in LR leads to reduced lipolysis rate, and consequently lower energy in blood vessel and brain. The latter triggers an increased gut input rate, which leads to the recovery of the fat compartment and of the immune response. rel., relative to homeostatic level.

As the model results identified fat as an important driver of immune responses, we investigated whether modulations of energy flow from fat to blood circulation would impact infection dynamics. We added lipolysis resistance (LR) *L*_r_ to the model (electronic supplementary material, equation S17) [54–57], where release of energy from fat is inhibited for values of *L*_r_ larger than zero. The effects of manually imposed LR are shown in figure 4. For acute infections, inhibition of lipolysis impaired pathogen clearance (figure 4*a,b*), which was associated with a reduction of fat energy (figure 4*c*). Sufficient LR turned the acute into a chronic infection.

In the setting of chronic infections, a sufficient inhibition of lipolysis resulted in pathogen clearance (figure 4*d*,*e*). The mechanisms underlying this counterintuitive effect, as implied by the model, involved the restoration of the fat compartment due to the orexigenic effect of reduced lipolysis (figure 4*j*). This beneficial effect of reduced lipolysis was offset by a long-term increase of fat energy associated with obesity [57] (figure 4*f*), energy deprivation from the brain (figure 4*g*) and the immune system (figure 4*h*) as well as high levels of fatty acids in the vessel compartment (figure 4*i*). These would lead to a deregulated state with impaired bodily functioning and accelerated long-term inflammation [43].

As a manual introduction of LR induced complex dynamics in the model, we next investigated the effects of dynamically regulated LR instead of a stepwise increase. The energy-flow model was extended by including an equation (electronic supplementary material, equation S18) for LR development, where *L*_r_ is driven by immune and stress response with rate *α*, and decays with rate *β*. The extended model is termed the LR model hereafter.

**Figure 5.**
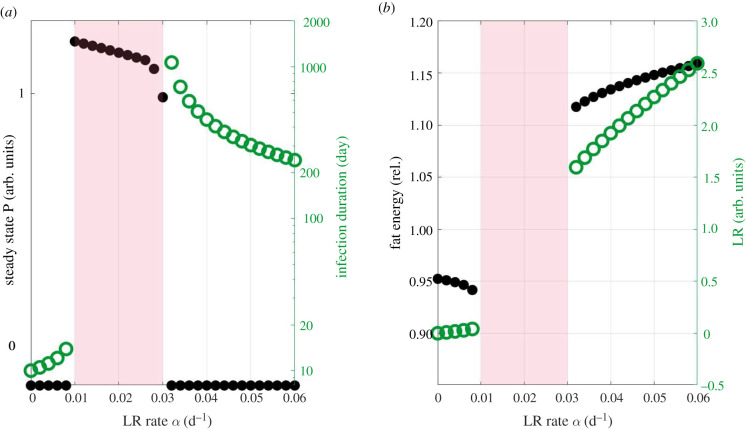
Dynamic lipolysis resistance (LR) impacts immune response to infections. *In silico* experiments in figure 2*a* were repeated using the LR model for different LR growth rates (*α*). (*a*) Steady state pathogen level (black dots, left axis) and infection duration (green circles, right axis) in simulations with clearance rate *d*_p_ = 1.16 d^−1^ (blue point in figure 2*a*). The infection duration is the time until the pathogen level dropped below 1% of the inoculation dose. (*b*) The amount of energy in the fat compartment (black dots, left axis) and the level of LR (green circles, right axis) at the time point of pathogen clearance (green circles in *a*). The light pink background indicates that the infection was not cleared within the simulated time frame, which is 100 years. Parameters in electronic supplementary material, table S1. rel., relative to homeostatic level; arb., arbitrary.

We investigated the impact of LR on infections by varying the LR growth rate *α*, while keeping the recovery rate fixed (*β* = 0.01 d^−1^). For an acute infection (i.e. *d*_p_ = 1.16 d^−1^), low LR growth rates did not change the outcome of the infection (figure 5*a*, black, left bottom), but the infection duration increased (figure 5*a*, green) from 10 to 20 days. At the time of pathogen clearance, fat energy decreased and LR increased with *α* (figure 5*b*, black and green, respectively). This suggested the existence of a vicious cycle: a longer infection duration providing more time for LR development, which in turn further impairs lipolysis and prolongs infection. There existed a critical LR growth rate, above which the pathogen persisted and the infection became chronic (figure 5*a*, middle part; see also electronic supplementary material, figure S5, for the corresponding bifurcation diagram). The observed impairment of the clearance of acute infections in the LR model confirms the model behaviour upon a manual stepwise LR increase (figure 4*a–c*).

LR-induced persistent infections only emerged for an intermediate range of *α* (figure 5*a*). With even larger LR growth rates *α*, the pathogen was cleared again (figure 5*a*, right bottom), albeit at a sluggish pace on a scale of hundreds of days (figure 5*a*, green). Fat energy and LR at the time of pathogen clearance were higher compared to acute infections (figure 5*b*, black and green, respectively). These results are consistent with the results of the manual stepwise LR in chronic infections (figure 4*d–j*).

## 3. Discussion

Energy is a dominant force for natural selection during evolution [58]. Immunity is subject to adaptive energy trade-offs during interplay with many physiological functions [20,31,59,60]. Although the idea of energy flow between compartments is a traditional scheme in physics and ecology [61], a comparable modelling approach that associated energy flow with interactions and functions of multiple organs is, to the best of our knowledge, a novel concept. The modelling approach presented here was constructed to describe energy allocation during an immune response to infections in the context of other functional compartments. The trade-offs between energy needs of different compartments emerged at the network level and gave rise to known associations of acute and chronic infections with collateral damage to the organism. Thus, we believe that the mathematical model presented in the language of energy flow can potentially explain manifold diseases and their pathogenesis from a transient versus permanent energy shortage in one compartment of the organism and related adaptations of other compartments.

A high pathogen clearance rate, corresponding to an efficient immune response, led to rapid pathogen removal (figure 2*a*) and subsequent return to healthy homeostasis (figure 2*b*). Given an extremely efficient immune response, the synergism arising from interactions with brain and fat tissue (electronic supplementary material, figure S2) would have been negligible and unnecessary. However, optimal protection by the immune system is rarely maximal [62], and higher clearance rates are also more likely to be associated with immunopathology (overshooting immune response and hyperinflammation) [63]. We therefore argue that most of the infections are cleared by a moderate clearance rate such that synergism due to brain and fat activities is crucial to achieve the two competing objectives: minimizing immunopathology and avoidance of chronic infections. *In silico,* chronic infections were associated with long-term low energy in brain and immune system (figure 3*d,e*), reflecting low performance in other vital functions, such as wakefulness/fatigue and immunocompetence.

The efficiency of the energy-flow system in supporting an immune response was quantified by the critical pathogen clearance rate (*C*_crit_) that separates acute from chronic infections. If it is low, the pathogen can be cleared by less aggressive immune responses associated with lower immunopathology. The mathematical model suggested that brain and fat tissue regulate the critical clearance rate, thus suggesting interesting targets for medical or behavioural interventions. We speculate that the health benefits associated with intermittent fasting [64] are related to its role in upregulating fat turnover (*L*_h_), which reduces the critical clearance rate (figure 2*e*).

The energy flow model presented here was strictly based on current knowledge of the inter-dependence of organismal compartments. While it also required several assumptions that should be tested in further studies, some simulation results, such as the typical duration of an acute infection (10 days, figure 2*a*) and fat loss in acute infections (5%, figure 3*f*), are consistent with common experience.

Further, the energy-flow model predicted that the synergism between the immune system and other tissues gives rise to a critical therapy initiation time, after which a treatment becomes ineffective (figure 2*g*). It has been recently established in clinical practice that for severe infections, like those of the bloodstream [65], severe bacterial infections [66], or sepsis [67], early instigation of antibiotic therapy (within 13.6 [67], 24 [66] or 48 [65] hours after diagnosis) is associated with reduced mortality. The reason for the observed critical time of antibiotic application will likely involve multi-organ and multi-level mechanisms. The consistency of simulated and clinical observation supports the usefulness of our energy-based perspective and serves to endorse any derived predictions.

The simulations suggested a timeline separating acute from chronic infections at around 18 days for the given parameter values (electronic supplementary material, figure S4). This timeline was also corroborated by the findings that in the context of therapy, the brain stress response was mainly supportive for immune responses before day 15, but suppressive for immune responses thereafter (figure 2*h,i*). This is in line with the observation that long-term activation of the brain stress axes can be harmful [48]. Interestingly, day 15 coincides with the peak of germinal centre reactions [68]. One might speculate that the emergence of high affinity antibodies as a last resort to fight infections triggers the switch from an elimination to a coexistence strategy.

However, the loss of fat energy appeared to dominate the acute–chronic division, due to its influence on both stress and immune responses. This is further supported by the predicted role of fat tissue LR or catecholamine resistance, which refers to the phenomenon that the lipolytic response of adipocytes to a sympathetic drive is attenuated [54–56]. This can happen through loss of *β*2-adrenoceptor signalling [56] or lack of local catecholamines [54,55] and is associated with ageing [55,69] and obesity [57,70]. Contrary to insulin resistance, which is considered a positively selected acute programme to deprive insulin-sensitive organs of energy to support the brain or immune system [71], LR impairs the flow of energy from fat tissue to circulation (the vessel compartment) and subsequently to the brain and immune system. It was anticipated that LR would delay the immune response to infection, which was confirmed by the model results (figure 4*a,b* and figure 5). However, overcritical inhibition of lipolysis induced clearance of otherwise chronic infection (figure 4*d,e* and figure 5) by its orexigenic effect. Longstanding LR has been associated with obesity [57] and reduced energy in brain and immune system (figure 4*f–h*), which may be associated with long-term changes such as inflamm-ageing and neurodegeneration. One might speculate that LR represents an additional layer of energy trade-off that is beneficial over the time scale of years but detrimental over longer periods. The slow regulation of energy release from fat might be critical in the development of chronic disease and accelerated ageing.

The mathematical model presented in this paper is subject to several limitations, such as the lack *inter alia* of a muscle compartment [52,72], daily feeding–fasting cycle and additional feedbacks in the stress response pathway as well as in the immune response pathway. Nevertheless, our energy-flow model enables investigation of the network of subsystems in diseases without the need for the explicit incorporation of complex molecular pathways. It considers the organism as a network of compartments interacting via competition for a limited resource—energy—and promises improved understanding of the mutual interdependence of subsystems as well as the development of new avenues of disease treatment with an eye on the whole organism. We hope these results will stimulate further collaborations between scientists in biology, physics and medicine, thus generating new ideas for the promotion of healthy ageing and disease intervention/management.

## Data Availability

The data are provided in electronic supplementary material [73].
